# Enhanced bone regeneration in rat calvarial defects through BMP2 release from engineered poly(ethylene glycol) hydrogels

**DOI:** 10.1038/s41598-024-55411-z

**Published:** 2024-02-28

**Authors:** Queralt Vallmajo-Martin, Christopher Millan, Ralph Müller, Franz E. Weber, Martin Ehrbar, Chafik Ghayor

**Affiliations:** 1 Department of Obstetrics, University Hospital Zürich, University of Zürich, Schmelzbergstrasse 12, 8091 Zurich, Switzerland; 2 School of Life Sciences and School of Engineering, Institute of Bioengineering, École Polytechnique Fédérale de Lausanne, Station 15, 1015 Lausanne, Switzerland; 3 Department of Urology, University Hospital Zürich, University of Zürich, Wagistrasse 21, 8952 Zurich, Switzerland; 4 Institute for Biomechanics, Eidgenössische Technische Hochschule Zürich, Leopold-Ruzicka-Weg 8093, 8049 Zurich, Switzerland; 5 Center of Dental Medicine, Oral Biotechnology & Bioengineering, University of Zürich, Plattenstrasse 11, 8032 Zurich, Switzerland

**Keywords:** Hydrogel, Osteogenesis, Bone regeneration, Bone defect, BMP2, Bioinspired materials, Biomaterials - cells, Regeneration

## Abstract

The clinical standard therapy for large bone defects, typically addressed through autograft or allograft donor tissue, faces significant limitations. Tissue engineering offers a promising alternative strategy for the regeneration of substantial bone lesions. In this study, we harnessed poly(ethylene glycol) (PEG)-based hydrogels, optimizing critical parameters including stiffness, incorporation of arginine-glycine-aspartic acid (RGD) cell adhesion motifs, degradability, and the release of BMP2 to promote bone formation. In vitro we demonstrated that human bone marrow derived stromal cell (hBMSC) proliferation and spreading strongly correlates with hydrogel stiffness and adhesion to RGD peptide motifs. Moreover, the incorporation of the osteogenic growth factor BMP2 into the hydrogels enabled sustained release, effectively inducing bone regeneration in encapsulated progenitor cells. When used in vivo to treat calvarial defects in rats, we showed that hydrogels of low and intermediate stiffness optimally facilitated cell migration, proliferation, and differentiation promoting the efficient repair of bone defects. Our comprehensive in vitro and in vivo findings collectively suggest that the developed hydrogels hold significant promise for clinical translation for bone repair and regeneration by delivering sustained and controlled stimuli from active signaling molecules.

## Introduction

The repair of large bone defects remains a major clinical challenge. Non-union fractures are associated with loss of bone function and severely impact the quality of life of the affected patients. Bone grafting remains by far the most frequently used technique to reconstruct large bone segments^1,2^. This technique generally leads to favorable clinical results, but it is associated with many drawbacks^3^. These include high socio-economic costs due to the extensive surgical procedures involved, and considerable numbers of postoperative morbidities and graft resorptions. Alternatives, especially for young patients where the availability of autologous bone graft material is limited, are needed^4^.

Bone tissue engineering approaches comprising novel biomaterials and stem cells have recently evolved as a promising strategy to aid, enhance or induce bone formation in critically-sized defects^5^. Biomaterials that serve as bone substitutes, often referred to as scaffolds, can assist natural healing via osteoconductive, osteoinductive, or osteogenic mechanisms^6^. Several biomaterials have already been explored for this application. Metal scaffolds such as titanium have been highly appealing due to their ability to bear load similar to bone tissue itself^7,8^. Unfortunately, these scaffolds have limited biological activity and often do not integrate properly with the native bone resulting in failed transplants^9^. Bioceramic scaffolds similarly sustain mechanical load while also allowing heightened host integration^10^. However, these materials do not allow abundant cell infiltration and remodeling of the construct into host bone tissue, often resulting in suboptimal regeneration. To overcome this, soft biomaterials like hydrogels, with properties suitable for the encapsulation of cells and the release of growth factors, have become promising alternatives^11,12^.

Different hydrogel materials have been used as matrices for bone tissue engineering and regeneration^13^. To enable optimal bone healing, ideal materials should exhibit mechanical stability, controlled degradability, integrin binding, and growth factor release to allow cell attachment, migration, proliferation, and ultimately differentiation of wound healing progenitor cells. Additionally, their properties should be tailored to ensure resorption of the material in concert with remodeling of the new bone^4^, and allow inosculation with the host tissue^14^. Hydrogels derived from natural components of the extracellular matrix (ECM), such as collagen or fibrin, have been widely used because of their physiological properties to aid in tissue regeneration^15^. Natural occurring hydrogels have shown to induce bone regeneration when loaded with supra-physiological concentrations of BMP2. Several alternatives have been successfully employed to augment bone regeneration by sustained BMP2 release. These strategies encompass the use of natural hydrogels combined with small mesh sizes^16^, the modification of hydrogels with native or engineered binding motives for growth factors like heparin^17^ or fibronectin^11^, and the integration of carriers for growth factors, such as nanogels^18^ or micro-particles^19^. Additionally, engineering BMP2 with heparin binding sites imparts a strong affinity for various ECM components, including collagen and fibrin, resulting in enhanced bone regeneration through the emulation of natural growth factor presentation^20^. Nonetheless, the challenges in customizing growth factor release and engineering the mechanical, physical, and biological characteristics of natural hydrogels have hindered their clinical translation^21^. Research, including our own^22^ as well as other studies^23^, has shown that naturally occurring materials are highly susceptible to proteolytic degradation leading to restricted bone formation as a result of reduced host cell remodeling.

Synthetic and semi-synthetic hydrogels can be tailored to mimic key in vivo microenvironments in simplified yet accurate models, which is crucial for successful tissue engineering approaches^24,25^. Therefore, in the field of regenerative medicine and tissue engineering, these hydrogels have undergone extensive investigation^26,27^. Poly(ethylene glycol) (PEG) is one of the most widely used polymers to form fully defined hydrogels. PEG hydrogels have significant advantages such as excellent biocompatibility and flexibility, but are associated with certain limitations such as poor mechanical strength and cell attachment. However, the limitations inherent to PEG as a biomaterial can be overcome by engineering approaches including the incorporation of cell adhesion sites and matrix metalloproteinase (MMP) sensitive crosslinks to allow cell infiltration and remodeling both in vitro and in vivo^24,28^. The integrin binding peptide Arg-Gly-Asp (or RGD) is frequently introduced as a functional group to promote the survival of adherent dependent cells such as osteoblasts^29^. In recent decades, PEGs have been extensively used for controlled delivery of growth factors and/or cells to promote tissue regeneration^12,27,30^.

We have previously developed modular PEG hydrogels designed to undergo enzymatic crosslinking by transglutaminase factor XIII (TG-PEG). Use of TG-PEG additionally enabled the immobilization of growth factors and cells owing to the specific and mild crosslinking conditions. Additionally, the modularity of the system allows for untethered tailoring of different individual hydrogel parameters to optimize for promotion of desired cell behaviors^31–33^. Indeed, TG-PEG has served as a valuable three-dimensional (3D) substrate to study the functions of human bone marrow derived mesenchymal stromal cells (hBMSCs) in vitro^32^.

In this study, we used TG-PEG hydrogels and examined the influence of different parameters such as stiffness, presence of RGD motifs, degradability, and the ability to deliver BMP2 to induce osteogenesis both in vitro and in vivo. The in vitro results show that the rigidity, the degradability and the presence of the RGD motifs influence the proliferation and the ECM deposition of cells. In addition, TG-PEG hydrogels continuously release incorporated BMP2 in its bioactive form, making them good candidates for in situ bone regeneration. Next, we performed a systematic in vivo investigation and found that TG-PEG hydrogels with low stiffness (200 Pa) enable faster regeneration of critically-sized defects of the rat calvarial bone compared to stiffer (400 and 1000 Pa) or softer (75 Pa) hydrogels. These optimal TG-PEG hydrogels were completely degraded and replaced by new bone after 4 weeks of implantation. In contrast, TG-PEG hydrogels with progressively increasing stiffness restricted cell infiltration and directed bone formation only towards their surface. These encouraging results demonstrate the potential of TG-PEG hydrogels for clinical translation in bone repair, where the stability of biomaterials and the sustained release of active signaling molecules must be in tune with bone regeneration.

## Results

### Tailoring TG-PEG hydrogel mechanical and physical properties

**Figure 1:**
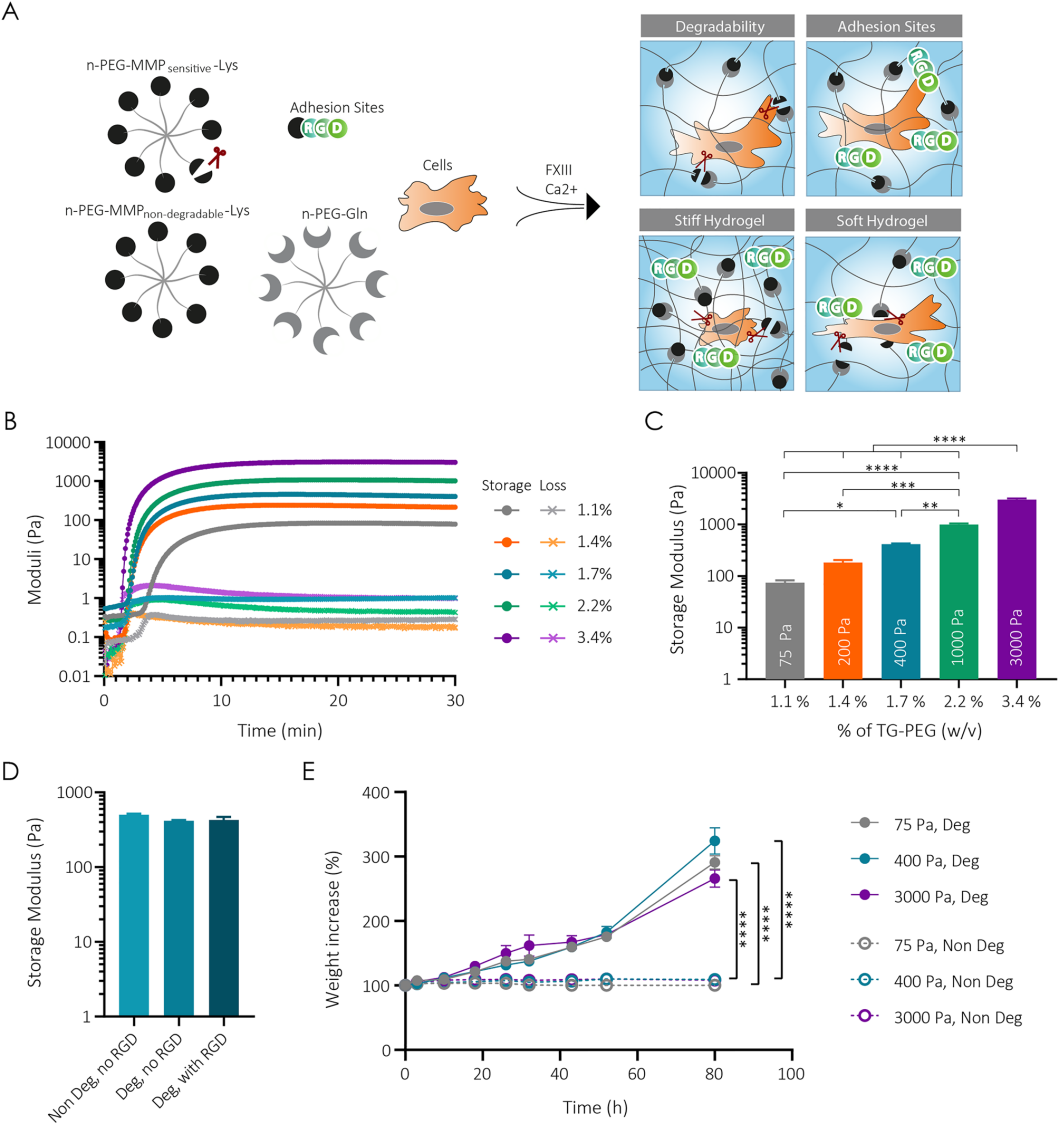
Mechanical and physical characterization of TG-PEG hydrogels. (**A**) Scheme of modular designed TG-PEG hydrogels. By combining different PEG backbone molecules and cell-adhesion peptide RGD, hydrogels with different degradability, cell adhesion properties and stiffness can be engineered and cross-linked in the presence of cells under physiological conditions. (**B**–**D**) Rheological characterization of TG-PEG hydrogels. (**B**) Representative mechanical profile consisting of storage modulus (G′, represented as dots) and loss modulus (G′′, represented as crosses) of TG-PEG hydrogels at different polymer concentrations ranging from 1.1% to 3.4% (w/v). Storage moduli of hydrogels after reaching a plateau at 30 min (**C**) at different polymer concentrations (n = 3) or (**D**) at a fixed 1.7% (w/v) polymer concentration containing MMP_sensitive_ (Deg) or MMP_non-degradable_ (Non Deg) cross-links, and with and without RGD adhesion sites (n = 3). (**E**) MMP1-mediated degradation over time of different percentages of TG-PEG hydrogels containing MMP_sensitive_ (Deg) or MMP_non-degradable_ (Non Deg) cross-links. Degradation of cross-links results in the swelling of hydrogels (n = 5). All data are reported as mean ± standard error. ANOVA with Tukey’s post hoc test *P < 0.05, **P < 0.01, ***P < 0.001, ****P < 0.0001.

With the goal of developing an optimal scaffold for healing large bone defects, we elected to work with a modularly designed synthetic biomaterial that is built from star-shaped 8-arm PEG precursors previously used in our lab (Fig. 1A)^34^. Functionalization of the PEG arms with either Gln-acceptor or a Lys-donor peptides resulted in 8-arm PEG-Gln and 8-arm PEG-Lys precursors, respectively. To render the PEG backbone degradable by cellular proteases, a Lys peptide containing an MMP-degradable sequence (MMP_sensitive_-Lys) was employed to form PEG-MMP_sensitive_-Lys. The formation of MMP-non-degradable or degradable TG-PEG hydrogel networks occurred through the factor XIII (FXIII) catalyzed polymerization of an equimolar mixture of PEG-Gln and PEG-Lys or PEG-MMP_sensitive_-Lys, respectively. During this polymerization, the TG-PEG hydrogel was functionalized using additional Lys-tagged cell adhesion ligand RGD (Lys-RGD). For the generation of three-dimensional (3D) cell constructs used in this study, hydrogel formation was conducted in presence of cells or growth factors.

To generate TG-PEG hydrogels with different levels of stiffness, precursor solutions with increasing initial polymer concentrations were formulated. Rheological measurements showed an inverse correlation of concentration and time to cross-linking, ranging from 1.5 min for hydrogels composed of 3.4% (w/v) PEG compared to 3.5 min for the 1.1% (w/v) hydrogel (Fig. 1B). Storage moduli were recorded 30 min after polymerization when a plateau was reached and revealed a concentration-dependent increase in hydrogel stiffness. Initial TG-PEG precursor concentration of 1.1% (w/v) resulted in hydrogels with storage modulus of 75 Pa (considered as very low stiffness), 1.4% in 200 Pa (considered as low stiffness), 1.7% in 400 Pa (considered intermediate stiffness), 2.2% in 1000 Pa and 3.4% in 3000 Pa (considered high stiffness) hydrogels (Figs. 1C, S1A and Table 1).

**Table 1:** Detailed storage moduli of each TG-PEG percentage (w/v) mix.

% (w/v) TG-PEG	Storage modulus (Pa)	Referred as (Pa)
1.1%	74.50 ± 14.72	75
1.4%	183.3 ± 37.87	200
1.7%	441.6 ± 62.58	400
2.2%	999.0 ± 73.78	1000
3.4%	3037 ± 270.2	3000

To investigate if incorporation of degradability or cell adhesion sites influences hydrogel stiffness, TG-PEG hydrogels of intermediate stiffness were formed with the non-degradable PEG-Lys or the PEG-MMP_sensitive_-Lys, and with or without 50µM Lys-RGD, respectively. Rheological evaluations of swollen hydrogels revealed equal stiffness (ca. 400 Pa) independent of the use of MMP-non-degradable sites and RGD (Figs. 1D, S1B).

Next, to test hydrogel susceptibility to proteolytic degradation, MMP-degradable and non-degradable hydrogels with different stiffnesses were incubated in the presence of 5 nM MMP1. Weight increase was monitored during incubation of hydrogels with the protease as degradation leads to additional uptake of water within the gels. We found that weight increase during degradation was similarly time-dependent for all hydrogels containing the MMP_sensitive_ crosslinks. In contrast, the non-degradable hydrogels (without MMP_sensitive_ moieities) did not change their weight over the course of the experiment indicating the absence of degradation (Fig. 1E). Together these data show that hydrogels with defined stiffness, degradability and cell adhesion properties can be formed by tailoring the composition of the initial precursor solutions.

### Optimizing TG-PEG hydrogels to serve as substrates for tissue growth and remodeling in vitro

**Figure 2:**
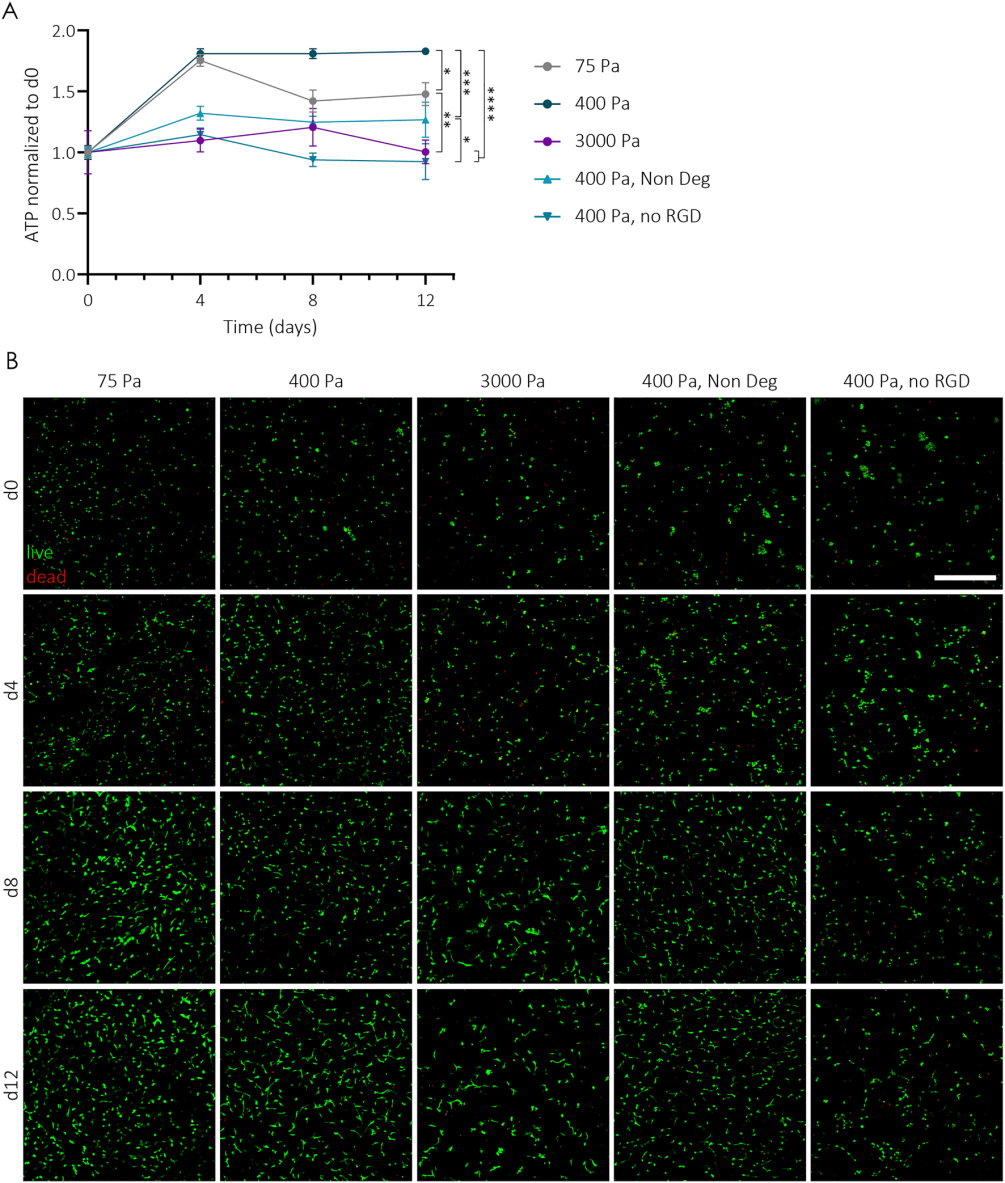
In vitro biological response of TG-PEG hydrogels. (**A**, **B**) Human bone marrow-derived stromal cells (hBMSCs) were encapsulated at 1.5 × 10^6^ cells per ml of TG-PEG hydrogels. TG-PEG hydrogels at different stiffnesses (75, 450 or 3000 Pa) containing MMP_sensitive_ degradable sites and RGD cell adhesion sites were tested, as well as TG-PEG gels at a fixed stiffness of 450 Pa lacking either the MMP_sensitive_ site (Non Deg) or the RGD peptide (no RGD). (**A**) Proliferation of encapsulated hBMSCs in the different hydrogels at days 4, 8 and 12 was assessed by ATP content and normalized to ATP content of hBMSCs at d0 right after encapsulation (n = 5). Data are reported as mean ± standard error. ANOVA with Tukey’s post hoc test *P < 0.05, **P < 0.01, ***P < 0.001, ****P < 0.0001. (**B**) Representative images of live and dead staining on hBMSCs over time encapsulated in different hydrogel conditions (live cells stained by calcein are in green, while dead cells stained in ethidium bromide are in red; scale bar: 500 µm).

To examine the influence on cell behaviors of the optimized hydrogels, hBMSCs were encapsulated in hydrogels with different properties and cultured for 12 days (Fig. 2). Proliferation of encapsulated cells was measured by quantifying ATP at various time points via CellTiter Glo assay. We observed that proliferation of hBMSCs was significantly higher in degradable hydrogels with intermediate stiffness that additionally contained cell adhesion sites (Fig. 2A). In stiff hydrogels, even in the presence of RGD, there was less proliferation than in their softer counterparts. hBMSC proliferation was significantly higher in very soft hydrogels in comparison to stiff gels, yet it was significantly lower than in the intermediate stiffness hydrogels. Additionally, a decrease in cell activity was observed in the 75 Pa hydrogels between days 4 and 8, which can be attributed to the cells degrading the hydrogels. This degradation led to a loss of structural integrity and stability of the soft hydrogel, prompting the cells to migrate into a monolayer culture on the plastic surface of the well plate. Interestingly, when RGD was missing from intermediate stiffness hydrogels, proliferation significantly dropped, and actually cells did not proliferate at all. Similarly, the absence of degradable sites also resulted in a significant decrease in hBMSC proliferation. These results were further corroborated by live and dead stainings of the whole hydrogels (Figs. 2B and S2).

**Figure 3:**
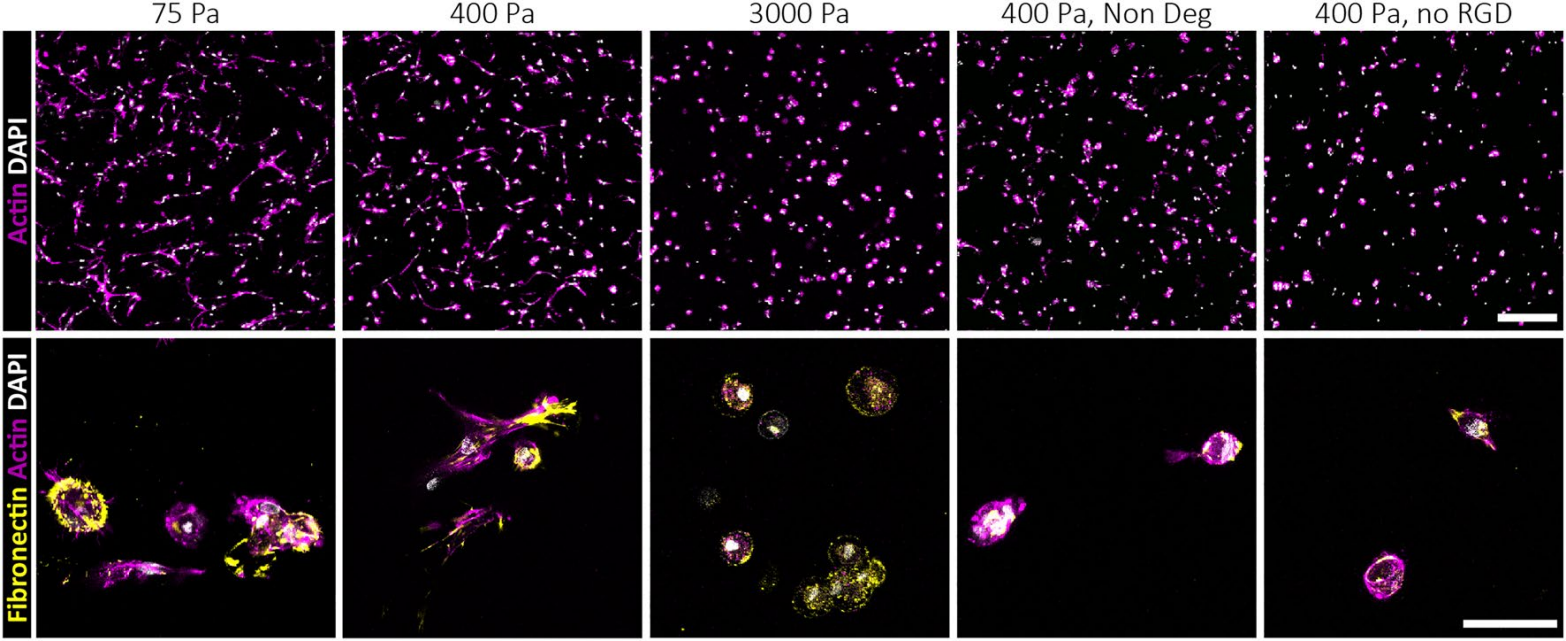
hBMSCs rapidly deposit ECM in synthetic TG-PEG. Representative images of hBMSCs 3 days post-encapsulation in different hydrogel conditions. Actin and DAPI staining displays hBMSC morphology (top; actin in magenta, DAPI in white; scale bar: 200 µm), while specific immunostaining for fibronectin reveals extracellular matrix deposition already 3 days post-encapsulation (bottom; fibronectin in yellow; scale bar: 50 µm).

Next, we used in-situ phalloidin staining of actin cytoskeletons to visualize cell spreading. Aligning with increase in proliferation, we observed that hBMSCs spread most when encapsulated in very soft and intermediate stiffness TG-PEG gels that additionally contained both degradable and RGD sites (Fig. 3 upper panel). At high stiffness, on the other hand, cell spreading was suppressed observed by lack of actin and round hBMSC morphology. The absence of RGD or degradable sites allowed the formation of minimal cell extensions, but still compromised cell spreading and cell network formation.

To remodel TG-PEG hydrogels into newly formed tissue, it is key that encapsulated cells can rapidly deposit their own ECM^35^. Fibronectin is a key ECM protein due to its high abundance and the fact that it permits cell spreading and deposition of other ECM proteins. So, we next evaluated the capacity of hBMSCs to deposit endogeneous fibronectin in various TG-PEG hydrogel conditions after 3 days of culture. hBMSCs rapidly deposited fibronectin in very soft and intermediate hydrogels only when MMP1-sensitive and RGD sites were available (Fig. 3 lower panel). Together, based on proliferation, spreading, and deposition of endogenous ECM proteins, intermediate hydrogels with degradable and cell adhesion sites were determined to be optimal substrates for hBMSC culture in vitro.

### BMP2 loaded TG-PEG hydrogels show sustained growth factor release over time

**Figure 4:**
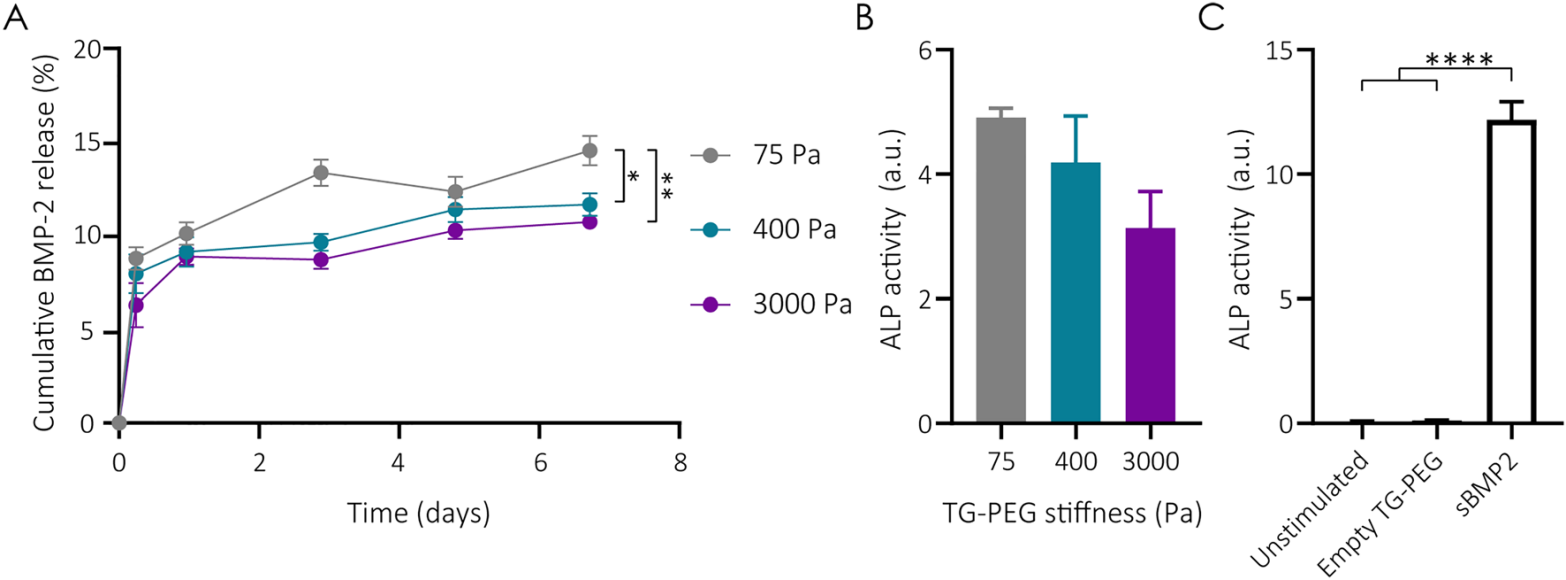
BMP2 release and activity from TG-PEG hydrogels. (**A**) Longitudinal release of BMP2 encapsulated in TG-PEG hydrogels at different stiffness measured by ELISA and calculated as the % of cumulative BMP2 released over total amount of BMP2 encapsulated (n = 4). (**B**) Relative ALP activity of C2C12 cells stimulated for 7 days by BMP2 being released from TG-PEG hydrogels (2 µg per gel) at different stiffnesses in a transwell setup (n = 3). (**C**) Controls of ALP quantification of C2C12 cells unstimulated (n = 3), or stimulated with an empty TG-PEG hydrogel (n = 3), or by direct addition of soluble BMP2 (2 µg per well, n = 3). All data are reported as mean ± standard error. ANOVA with Tukey’s post hoc test *P < 0.05, **P < 0.01, ****P < 0.0001.

Another key aspect for biomaterial design to aid tissue regeneration is their ability to release biologically active growth factors. To test the ability of TG-PEG hydrogels from different stiffnesses to release BMP2, we loaded very soft, intermediate and stiff TG-PEG hydrogels with 500 ng of BMP2. Hydrogels were then transferred into a wash solution, and supernatants were longitudinally collected at different time points for 7 days. Subsequently, BMP2 concentration was measured by ELISA (Fig. 4A). In the first 24 h, all TG-PEG-hydrogels had an initial burst release of BMP2. For the intermediate and high stiffness hydrogels, the release plateaued after 24 h. In contrast, the cumulative release of BMP2 from very low stiffness hydrogels followed a significant increase until the third day before stabilizing. At the end of the followed time course, overall BMP2 release was significantly higher in very soft hydrogels (72.68 ± 7.96 ng) compared to their stiffer counterparts (58.26 ± 6.02 ng for 400 Pa hydrogels and 53.61 ± 3.75 ng for 3000 Pa). In addition to the importance of observing BMP2 release from the gels, we assessed the bioactivity of the released growth factor to ensure it still retained its osteogenic potential. To investigate BMP2 bioactivity, we evaluated the osteogenic differentiation of the myoblastic cell line C2C12 after being stimulated with BMP2-laden TG-PEG hydrogels from different stiffnesses. C2C12 cells, unlike primary-sourced cells which are intrinsically heterogenous such as hBMSCs, serve as a standardized cell line to test BMP2 activity. C2C12 cells, upon BMP2 stimulation, differentiate towards osteogenic lineage, change their morphology from spindle shaped to a cuboidal morphology, and increase the expression of ALP in an almost linear dose response curve^36^. Hydrogels were prepared in a transwell insert, such that the continuously released BMP2 diffused into the medium of the cultured C2C12 cells. The observed alkaline phosphatase (ALP) activity, an early marker of osteogenic differentiation, clearly showed the osteogenic differentiation of C2C12 cells in response to BMP2 releasing TG-PEG hydrogels of all stiffnesses (Fig. 4B and controls in Fig. 4C). Noteworthy, osteogenic differentiation in C2C12 cells as measured by ALP activity was greater for conditions in which BMP2 was released from the softest TG-PEG hydrogels as opposed the lowest observed ALP activity in stiffer hydrogels. This corroborates well with the findings that very soft hydrogels released significantly higher amounts of BMP2. Overall, these results reflect hydrogel stiffness-dependent dynamics of BMP2 release and confirm the bioactivity of the released growth factor.

### TG-PEG hydrogels loaded with BMP2 induce bone regeneration in a calvarial defect model

**Figure 5:**
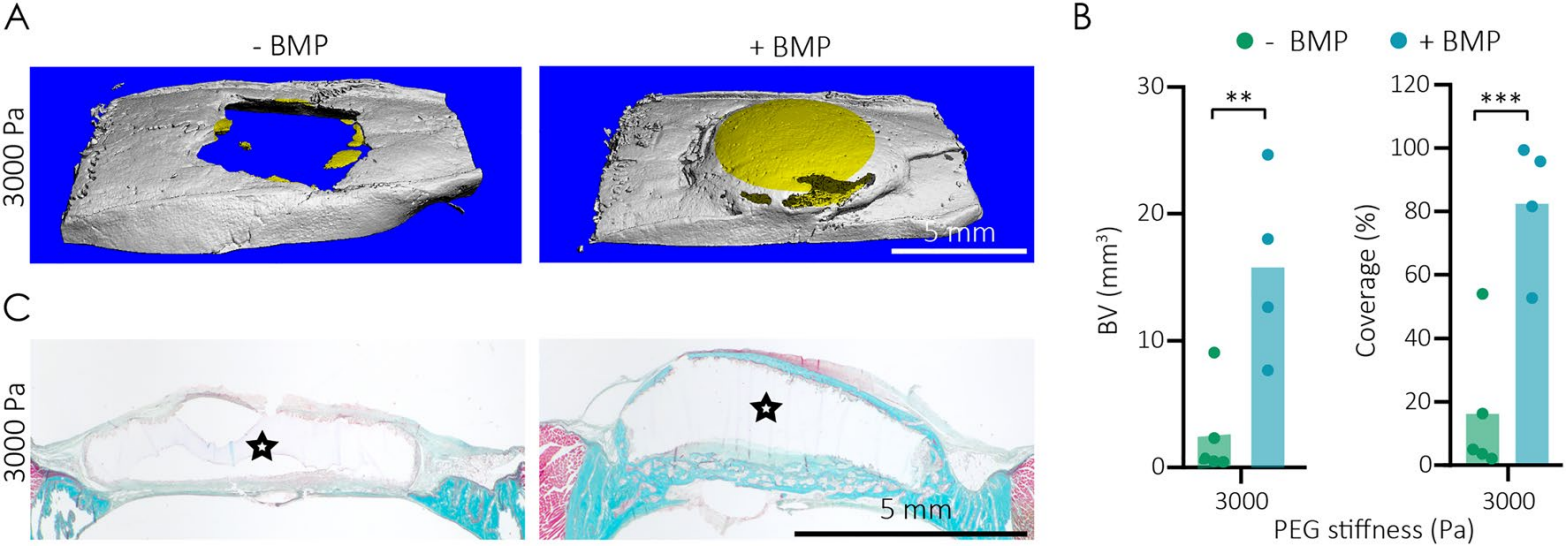
TG-PEG hydrogel loaded with BMP2 induces bone regeneration in vivo. (**A**–**C**) Healing of calvarial defects over four week treatment with control (0 µg) or BMP2 loaded (5 µg per gel) stiff TG-PEG hydrogels (3000 Pa). (**A**) Representative microCT reconstruction of treated calvarial defects. Native bone in gray and treatment site in yellow. (**B**) MicroCT quantifications of bone volume and coverage (n > 4). All data are reported as individual data points and means. ANOVA with Tukey’s post hoc test ** P < 0.01, *** P < 0.001. (**C**) Representative Goldner trichrome stained histological section through the middle of the treatment site. Bone tissue in magenta; remaining TG-hydrogel labelled with stars.

The efficiency of bone regeneration is strongly dependent on the sustained delivery of suitable and bioactive growth factors^27,37^, as well as on the provision of a three-dimensional matrix that balances stability for host cell invasion with its ability to undergo remodeling^38^. Indeed, our thorough in vitro evaluations revealed that the modular-designed TG-PEG hydrogel is an excellent platform to independently tailor individual parameters for this purpose. Next, to establish parameters relevant for bone regeneration, we employed a critical size (8 mm diameter) rat calvarial bone defect model. This flat bone defect is ideal for the reproducible testing of equally shaped hydrogels independent of any load-bearing stimuli. To provide sufficient in vivo stability and stiffness for bone regeneration, we first implanted TG-PEG hydrogels of 3000 Pa that contained 0 or 5 µg of BMP2, a BMP2 concentration comparable to one previously used in critically sized rat femoral segmental defects^39^. After 4 weeks of implantation, microCT analyses were performed to assess if these BMP2-releasing TG-PEG hydrogels could direct bone regeneration. Thresholded microCT images of the control TG-PEG hydrogel showed only very small bone particles at the edge of the defect, while the BMP2-releasing hydrogels were almost completely remodeled with new bone (Fig. 5A). Quantifications revealed significantly higher bone volume (BV) and bone coverage in the defect area when hydrogels contained BMP2 compared to controls (Fig. 5B). Histological evaluations of the defects treated with BMP2-releasing TG-PEG hydrogels confirmed that bone was only formed on the periphery of the implanted biomaterial (Fig. 5C). In both control and BMP2-releasing hydrogels the host cells infiltrated the hydrogel to a very minimal extent. Together, these data show that stiff TG-PEG hydrogels, despite their ability to release bioactive BMP2, induce the apposition of new bone relative to the tissue engineered scaffold rather than regeneration of the whole bone defect.

### TG-PEG hydrogel stiffness determines the distribution of regenerated bone

**Figure 6:**
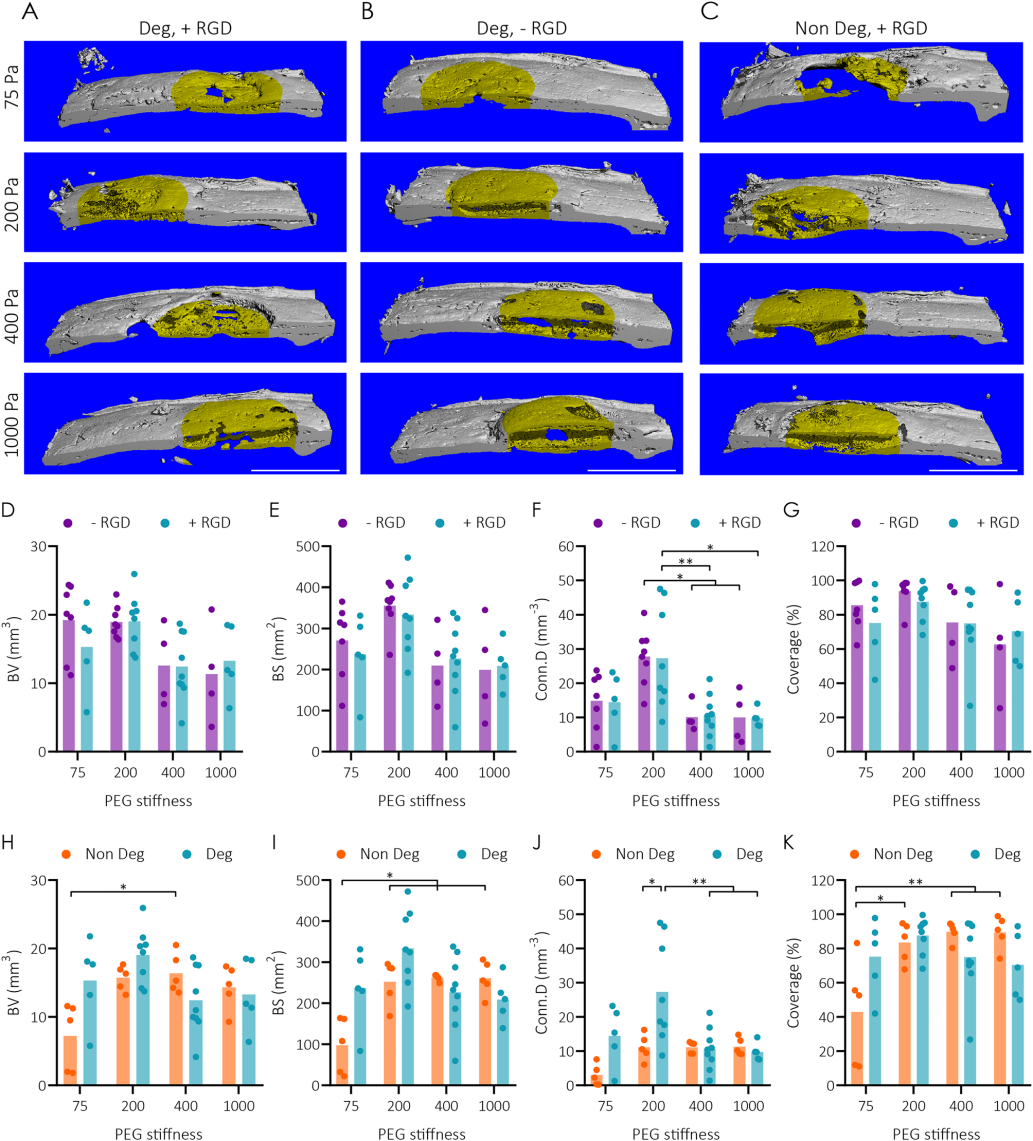
Tailoring BMP2-delivering-TG-PEG hydrogels properties to enhance bone regeneration in rat calvarial defects. Healing of calvarial bone defects by for four week treatment with BMP2 loaded TG-PEG hydrogels of different stiffnesses that contained or not 50 µM RGD, and that contained MMP_sensitive_ (Deg) cross-links or MMP_non-degradable_ (Non Deg). (**A–C**) Representative microCT reconstruction of treated calvarial defects with (**A**) hydrogels containing MMP_sensitive_ and RGD sites, (**B**) hydrogels containing MMP_sensitive_ and no RGD sites, and (**C**) hydrogels containing MMP_non-degradable_ and RGD sites (scale bars = 5 mm). Native bone in gray and treatment site in yellow. (**D–G**) MicroCT quantifications comparing MMP_sensitive_ hydrogels containing or not RGD sites based on (**D**) bone volume (BV), (**E**) bone surface (BS), (**F**) connectivity (Conn.D), and (**G**) coverage (n > 4). (**H–K**) MicroCT quantifications comparing RGD containing hydrogels with MMP_sensitive_ or MMP_non-degradable_ cross-links based on (**H**) bone volume (BV), (**I**) bone surface (BS), (**J**) connectivity (Conn.D), and (**K**) coverage (n > 4). All data are reported as individual data points and means. ANOVA with Tukey’s post hoc test * P < 0.05, ** P < 0.01.

Lack of bone formation within the hydrogel and the absence of cell infiltration indicated that the preliminary hydrogel formulation described above could be further tailored to obtain more complete bone regeneration. Stiffness, the presence of cell adhesion sites, and hydrogel degradability are parameters that critically influence infiltration of the hydrogel by cells in vitro and, likely, also in vivo. Since infiltrating host cells need to locally degrade hydrogel cross-links to facilitate penetration into the gel, we reasoned that lowering the stiffness (i.e. lower crosslinking density) of TG-PEG hydrogels might be the most effective in accelerating hydrogel degradation by infiltrating host cells in vivo. And that, in turn, might allow host cells to deposit endogenous ECM most effectively, resulting in bone regeneration. To test this, various BMP2-releasing TG-PEG hydrogel formulations ranging from 75 to 1000 Pa stiffness, and that contained both MMP-degradable sites and RGD were compared (Fig. 6A). While microCT-based imaging demonstrated bone apposition on the surface of the stiffest hydrogels of 1000 Pa, in hydrogels with lower stiffness we observed bone formation towards the center of the original hydrogels. Importantly, in hydrogels with very low and low stiffnesses (75 and 200 Pa) new bone was observed throughout the whole thickness of the defect, indicating that the TG-PEG hydrogel was completely remodeled within this timeframe.

To further characterize the regenerated bone, microCT data was quantified regarding the volume (BV) and the surface area (BS) of the regenerated bone as well as a parameter for the interconnectivity of measured bone areas (Conn.D) and the coverage of the bone defect (Fig. 6D–G, blue bars). While decreasing the hydrogel stiffness did not result in a significant change in BV, BS or coverage, a trend towards higher BV and BS could be observed in 200 Pa hydrogels. Conn.D showed a significant increase in soft (200 Pa) hydrogels as compared to intermediate (400 Pa) or stiffer (1000 Pa) hydrogels. This effect was lost for the very soft (75 Pa) hydrogels.

**Figure 7:**
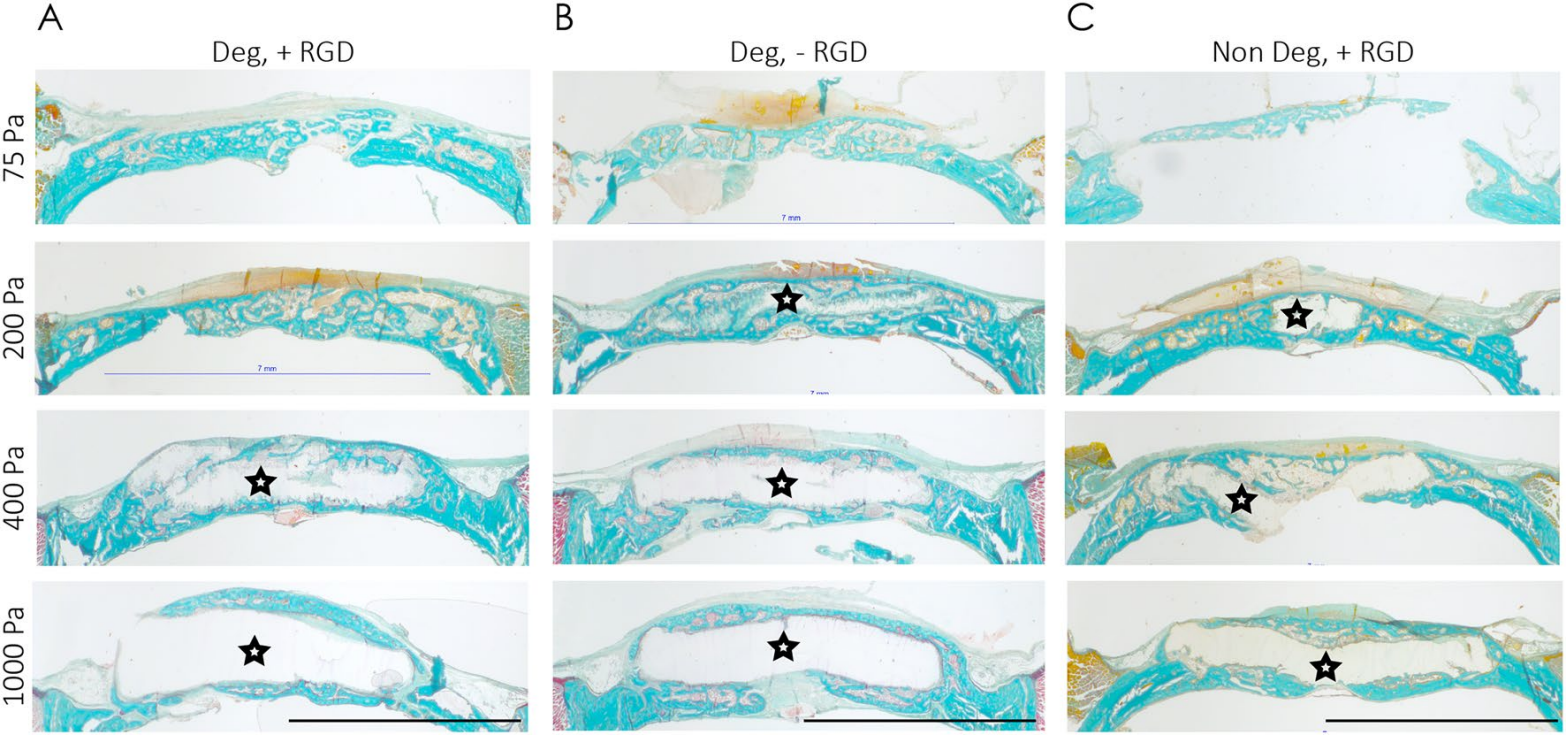
Influence of MMP degradability and cell adhesion sites in BMP2 releasing TG-PEG hydrogels on in vivo bone regeneration. (**A–C**) Representative Goldner trichrome stained histological sections through the middle of the treatment site. Defects were regenerated for 4 weeks with BMP2 loaded TG-PEG hydrogels of different stiffnesses containing (**A**) MMP_sensitive_ and RGD sites, (**B**) MMP_sensitive_ and no RGD sites, and (**C**) MMP_non-degradable_ and RGD sites (scale bars = 5 mm). Bone tissue in magenta; remaining TG-hydrogel labelled with stars.

Histological examination of Goldner trichrome stained tissue sections of the healed bone areas corroborated these microCT evaluations (Fig. 7A). They showed that the very soft and soft hydrogels (75–200 Pa) were completely replaced by new bone tissue, whereas hydrogels with intermediate stiffness were only partially remodeled and hydrogels with high stiffness remained mostly intact, with some signs of surface erosion.

### RGD is not required for in vivo bone regeneration by TG-PEG hydrogels that release BMP2

Cell attachment and migration depends on interactions between integrins and their ligands, which are abundantly present in native extracellular matrix proteins. In the TG-PEG hydrogels investigated here, the incorporation of the cell adhesion site RGD was shown to be necessary to support these integrin interactions in vitro. Therefore, we reasoned that hydrogels lacking RGD might significantly reduce infiltration by host cells and hamper their ability to proliferate and/or produce endogenous ECM, as seen in vitro. To test this hypothesis, we compared bone formation in hydrogels with or without RGD following transplantation in defects of the rat calvarial bone. Surprisingly, in our microCT evaluations, we saw no significant difference in bone formation between hydrogels containing or not containing RGD (Fig. 6A, B and D–G). In the histological evaluation of the treated defect sites, a slightly delayed infiltration of hydrogels was observed in hydrogels that did not contain RGD versus hydrogels with RGD (Fig. 7B). These observations show that the presence of RGD in the tailored hydrogels does not play a major role in the regeneration of bone defects.

### Degradable soft TG-PEG hydrogels allow complete bone regeneration

The rate of scaffold degradation and the release of growth factors are important and likely interconnected factors in tissue regeneration after scaffold implantation. Therefore, next, we examined the influence of PEG-hydrogel degradability on rate of bone regeneration. For this, BMP2 loaded degradable and non-degradable TG-PEG hydrogels with different stiffnesses were tested in the rat calvarial bone defect. MicroCT analyses revealed that less bone had formed during the 4‐week period when defects were treated with non-degradable compared to degradable hydrogels (Fig. 6A and C). This difference was most obvious in the softest hydrogel. Also, the degradable soft (200 Pa) TG-PEG hydrogel performed better in terms of BV and BS than the non-degradable counterparts. Importantly, for Conn.D, the performance of the degradable hydrogel was significantly better than the non-degradable counterpart (Fig. 6H–K). Histological studies further corroborated these findings (Fig. 7C). In stiff degradable or non-degradable hydrogels, differences in cell invasion could not be detected. However, in intermediate stiffness hydrogels, the infiltration appeared to be decreased in non-degradable gels. At low and very low stiffness, the presence of degradable sites resulted in more efficient remodeling of the hydrogels and faster regeneration of bone. Together these data show the need for degradable sites in BMP2 delivering hydrogels to achieve a fast and efficient bone regeneration.

## Discussion

Strategies to enable the regeneration of large bone defects through the mobilization of host cells and their gradual osteogenic differentiation hold significant importance. In this study, we investigated the influence of specific biomaterial properties on bone repair, capitalizing on the inherent regenerative capabilities of bone tissue. To accomplish this, we employed PEG-based hydrogels in which different attributes such as stiffness, cell adhesion sites, degradability, and BMP2 release capacity could be tailored individually. Our rationale was to systematically optimize these parameters, allowing us to dissect their respective impacts on cell accessibility, mobility, and behavior within the biomaterial constructs.

We have shown that it is possible to make TG-PEG hydrogels with different degrees of stiffness, ranging in storage moduli from 75 to 3000 Pa. Comparable to our previous study^34^, this was achieved by simply modifying the PEG concentration, which is related to the degree of cross-linking and is an indication of hydrogel’s capacity to store deformation energy in an elastic manner. Importantly, the addition of cell adhesion sites at cell culture-relevant (50 µM) concentrations and the integration of crosslinking sites susceptible to MMP-degradation did not significantly influence the hydrogel’s mechanical properties. Therefore, depending on the bone tissue we want to regenerate and the time available to carry out this action, it seems plausible to tailor this hydrogel by rationally engineering their stiffness, cell adhesion, and degradation properties.

A fundamental role of any scaffold is to provide the structural support and to guide tissue regeneration, with the ultimate goal to be completely replaced by the newly formed tissue. In order to mimic the behavior of host progenitor cells recruited into our hydrogel, we conducted in vitro experiments to examine the spreading and growth of hBMSCs encapsulated within TG-PEG hydrogels. Through the incorporation of RGD motifs and MMP1-sensitive sites, our in vitro studies demonstrated that hydrogels with very low (75 Pa) and intermediate stiffness (400 Pa) levels effectively promoted the spreading of encapsulated hBMSCs. Notably, hBMSCs solely proliferated in low (1.48 ± 0.21 fold) and intermediate stiffness (1.83 ± 0.06 fold) hydrogels, while they failed to proliferate in the absence of adhesion or degradation sites, as well as in high stiffness hydrogels (3000 Pa). These findings are in line with our previous research and those of others, demonstrating that synthetic hydrogels created through various cross-linking approaches support increased cell growth when integrin binding and degradation sites were present, particularly when they exhibited low stiffness^35,40–42^. Remarkably, even in optimal conditions, hBMSCs exhibited mild proliferation in synthetic TG-PEG hydrogels. These observations align with our previous research, where we demonstrated that hBMSCs encapsulated in TG-PEG hydrogels exhibited limited proliferation under control conditions but displayed significantly high proliferation when exposed to platelet-derived growth factor (PDGF-BB)^12^. This is not surprising, as synthetic hydrogels, unlike natural materials, lack inherent biological activity to directly stimulate cell proliferation. However, in agreement with previous data^35,43^, here we show that, even in the absence of growth factor stimulation, hBMSCs were able to establish their microenvironment by degrading the hydrogel through MMP activity and rapidly depositing their own ECM.

To generate a biomaterial with osteoinductive function, we chose to incorporate BMP2 to our hydrogel platform to recruit host cells and provide osteogenic signaling for their differentiation enabling production of bone-specific ECM. Our in vitro results with C2C12 cells, a cell line commonly used to test BMP2 activity^36^, showed that the bioactivity of BMP2 was maintained during release from our TG-PEG hydrogels. Interestingly, we found that an increase in the stiffness of hydrogels is linked to a reduction in burst release, suggesting the possibility of tuning BMP2 release by the adjusting the crosslinking density of this hydrogel. This aligns with previous studies showing that growth factor release can be controlled with varying substrate pore size^44^. However, in vitro release properties cannot fully recapitulate the effective in vivo release profiles due to the absence of crucial factors like host cell-mediated hydrogel degradation and inflammatory responses, which can significantly impact BMP2 delivery kinetics by affecting hydrogel stability and diffusion conditions. To further improve bone regeneration, active BMP2 could be released in an ECM-mimicking manner^45,46^. In our hydrogel system this was previously achieved by the integration of a streptavidin linker and the use of biotinylated BMP2 and has been shown to enhance bone regeneration^12,47^. Alternatively, conjugating glycosaminoglycans such as heparin to biomaterials was shown to prolong the release of BMP2 and thereby promote bone healing in vivo^48^.

In a previous longitudinal study on bone regeneration, we demonstrated that TG-PEG hydrogels delivering BMP2 achieved nearly complete coverage of murine calvarial bone defects within four weeks following implantation^49^. Our observations revealed that de novo bone formation predominantly occurred during the initial four weeks, coinciding with active bone healing and mineral deposition. Subsequently, in the later weeks, spanning from week 6 to 12, a distinct phase of bone remodeling was evident, involving the refinement of the initial bone structure. Therefore, in the present study, we anticipated that a 4-week treatment duration would reveal significant biomaterial-related disparities in cell infiltration and the initial localized bone formation, thus providing insight into optimizing healing responses. Longer treatment periods, conversely, would better capture the remodeling phase of newly formed bone and shield the early interactions between mobilized osteogenesis-mediating cells and the employed biomaterials.

Through our in vivo experiments, we have established that BMP2-free hydrogels, regardless of their chosen stiffness, do not promote the healing of critical bone defects within the 4-week evaluation period tested here. However, the introduction of BMP2 (5 μg/defect, equivalent to 118 μg/ml of hydrogel) resulted in robust bone formation within the defect model. These results are consistent with our previous findings, where PEG hydrogels containing similar BMP2 concentrations (100 μg/ml) efficiently regenerated murine calvarial bone defects, while lower concentrations (20 μg/ml) resulted in minimal bone regeneration^49^. These outcomes are also in line with the performance of protein-based hydrogels and other synthetic materials previously employed for bone regeneration^50^. Notably, higher BMP2 concentrations (200 μg/ml) were required to achieve efficient bone regeneration in critically sized rat femoral segmental defects when treated with alginate hydrogel or collagen sponges^39^. However, it is worth mentioning that protein-based hydrogels, such as collagen and fibrin, are susceptible to proteolytic degradation and may not provide the necessary stability for optimal bone regeneration, as demonstrated in previous studies^22^. Hence, the modular design of the TG-PEG hydrogels described in this study present an ideal platform for the independent optimization of individual biomaterial properties and their impact on bone regeneration^22,51^.

The evaluation of hydrogel stiffness revealed that very soft and soft hydrogels (75 Pa and 200 Pa) resulted in higher bone volume and bone surface compared to stiff hydrogels. The degradability of these hydrogel is dependent on both the cross-linking density of the hydrogels and the presence of degradation sites that facilitate cell-directed degradation via MMP activity. In vivo, we observed that the degradation kinetics were sufficiently rapid to support bone regeneration in the case of softer MMP-degradable hydrogels with stiffness levels of 75 and 200 Pa. However, even in the presence of MMP motifs, hydrogels with a stiffness of 1000 Pa did not fully degrade. It is worth noting that hydrogels with higher stiffness led to recruited host cells forming a surface apposition of newly formed bone, rather than infiltrating the hydrogel. This is likely due to the difficulty of cells to penetrate and remodel stiff hydrogels, as previously reported for PEG hydrogels containing different minimal plasmin-sensitive peptides^52^. In the case of stiffer gels, host cells likely remain on the surface, where, under the influence of released BMP2, they undergo differentiation into osteoblasts. It is of paramount importance that future experiments assess bone formation in stiffer hydrogels at later time points, such as 12 weeks post-implantation, as it is conceivable that stiff hydrogels, due to their slow degradation, may result in increased bone formation in a more protracted context. Furthermore, the use of leaching particles^53^ or the assembly of microgels^54^ could be promising strategies to decouple hydrogel stiffness and porosity.

In contrast to stiffness and degradation, the presence of cell adhesion sites had only minor effects on in vivo bone regeneration. This is in agreement with previous findings showing that cells deposit their own ECM when encapsulated into biomimetic hydrogels and, especially at high cell density, are not dependent on presence of cell adhesion sites provided by biomaterials themselves^30,55^. Similarly, the hydrogel’s MMP-susceptibility had no significant effect on bone regeneration. This seems to be in stark contrast to the limited ability of BMSCs to spread and migrate when encapsulated in non-degradable low density hydrogels in vitro. However, in in vivo*,* different proteases are involved in the biomaterial remodeling. Additionally, the infiltration of biomaterial is the result of a high cell number being present at the hydrogel interface. These findings clearly show that the testing of properties critical to design of a novel biomaterial in vivo is of great importance.

Taken all together, we anticipate that our findings will impact the bone tissue regeneration strategies currently available. Follow-up experiments to aid clinical translation will include testing the optimized conditions in a defect model of a long bone. A limitation of the flat bone models, such as calvaria, is the lack of load bearing^56^. We envision translating these findings to bigger animal models, such as sheep, where long bone defects much better represent the clinical situation, as other studies have shown^57^.

While hydrogels often cannot bear high loads, they facilitate remodeling into newly formed tissue by inducing or conducting delivered or infiltrated cells to mineralize and deposit bone extracellular matrix. Combinations of load bearing scaffolds with hydrogels that allow the delivery of BMP2 have shown promising results in the regeneration of rat calvaria bone defects^58^. However, formulations with optimized mechanical, structural and biological properties for potential clinical applications need to be developed. To sustain load-bearing situations, the presented TG-PEG hydrogels will likely need to be combined with other materials such as metal scaffolds or bioceramics. Bioceramic scaffolds include the widely used hydroxyapatite tricalcium phosphates (HA-TCP) which have been shown to efficiently induce bone ingrowth by osteoconduction and osteoinduction by changing their physicochemical properties and the architecture of the scaffold. For example, our group has recently shown that hydroxyapatite (HA) scaffolds produced by additive manufacturing with pore and bottleneck sizes between 0.7 and 12 mm best support the bridging of bone defects and regenerated bony area^59^. Additionally, when formed with identical pore architectures, processing techniques, and porosities of up to 40%, HA-based scaffolds promoted osteoconduction much more efficiently than tricalcium phosphate (TCP)-based scaffolds^60^. Thus, combining the architectural and mechanical properties of bioceramics with the cell-inducing-remodeling environment that soft BMP2 releasing TG-PEG hydrogels offer is an appealing approach to both regenerate the bone defect and bear loads during the regeneration process.

## Conclusions

We systematically tailored synthetic hydrogels to optimize bone formation in a calvarial bone defect model by engineering specific parameters such as stiffness, degradability, and cell-adhesion sites. A delicate equilibrium between scaffold stability and cell infiltration was successfully achieved in TG-PEG hydrogels with a stiffness of 200 Pa. Notably, when combined with BMP2, these hydrogels prompted robust mineralization and robust bone formation across the entire defect site. Strikingly, cell adhesion sites exhibited minimal influence on bone formation, while enabling cell-mediated degradation of the hydrogels significantly enhanced the process, facilitating complete remodeling of the injured area. This platform offers precise control over the conditions necessary for regenerating critically-sized bone defects and lays the foundation for the future development of material-based therapeutics tailored to personalized tissue engineering.

## Material and methods

All the methods were carried out in accordance with relevant guidelines and regulations.

### TG-PEG precursor synthesis and preparation

The synthesis of TG-PEG precursors was conducted as previously described^34,61^. Peptides (Bachem AG; purity of > 95%) consisting of a cysteine cassette (ERCG) and a factor XIII (FXIII) glutamine acceptor substrate sequence (Gln; H-NQEQVSPL-ERCG-NH_2_) or lysine donor substrate that is matrix metalloproteinase-degradable (MMP_sensitive_-Lys; Ac-FKGG-*GPQGIWGQ*-ERCG-NH_2_) or non-degradable “ND” (MMP_non-degradable_-Lys; Ac-FKGG-GDQGIAGF-ERCG-NH_2_) were used to functionalize 8-arm PEG-VS (PEG-vinylsulfone, 40 kDa MW; NOF). For this functionalization, a 1.2 molar excess of peptides and PEG-VS were dissolved separately in triethanolamine (TEA) at pH 8.0, mixed and then incubated for 2 h at 37 °C. After excessive dialyzing using dialysis tubes (3.5 kD cutoff) in pure water, the resulting purified 8-PEG-Gln and 8-PEG-MMP_sensitive or non-degradable_-Lys precursors were stored at – 20 °C. FXIII (200 U/ml, Fibrogammin P, CSL Behring), the transglutaminase needed to initiate hydrogel cross-linking, was activated by the reaction with thrombin (2 U/ml) for 30 min at 37 °C. The resulting FXIIIa was stored in small aliquots at – 80 °C until use.

### 3D TG-PEG hydrogel formation

8-arm PEG-Gln and 8-arm PEG-MMP_sensitive_-Lys or PEG-_non-degradable_-Lys were stoichiometrically mixed in Tris buffer (50 mM, pH 7.6) with calcium chloride (CaCl_2_, 50 mM). Different w/v % of TG-PEG precursors was used to accomplish different hydrogel stiffness. Additionally, 50 µM Lys-RGD peptide (Ac-FKGG-RGDSPG-NH_2_) was added to the precursor solution when cell adhesion sites were desired. To initiate cross-linking, 10 U/ml of activated transglutaminase factor XIII was added to the hydrogel mix. After vortexing, hydrogel mix was pipetted and sandwiched between two hydrophobic glass slides (treated with SigmaCote) resulting in disc-shapes. Gels were then incubated for 30 min at 37 °C in a humidified atmosphere at 5% CO_2_. Last, hydrogels were carefully released from the slides and directly used for in vivo transplantation, or transferred to tissue-culture well plates for in vitro experiments.

### In situ* rheometry*

Hydrogel stiffness was characterized by in situ rheometry, as previously described^22^. Briefly, 80 μl hydrogels at the indicated final dry mass content were precisely loaded onto the center of the bottom plate of a rheometer (MCR 301, Anton Paar) equipped with 20 mm plate–plate geometry (PP20, Anton Paar) at 37 °C in a humidified atmosphere. Next, the upper plate was lowered to a measuring gap size of 0.2 mm, and the dynamic oscillating measurement began. Analysis were performed at a constant angular frequency of 1 Hz and constant shear strain of 4%, corresponding to the linear viscoelastic region. The storage (G′) and loss modulus (G′′) was recorded for 30 min when equilibrium was reached.

### Degradation of gels by MMP1

TG-PEG hydrogels (15 μl) of different stiffnesses and with or without the MMP1_sensitive_ moiety were immersed in 100 μl digestion buffer (50 mM Tris, 50 mM NaCl, 10 mM CaCl_2_, 0.05% (w/v) Brij 35, pH 7.5) containing 5 nM MMP1 (PeproTech). The weight of hydrogels was measured repeatedly during incubation at 37 °C until degradable gels were digested.

### Growth factor release in vitro

TG-PEG hydrogels (10 µl) of different stiffness containing 0.5 µg of BMP2 were incubated in 200 µl of 0.1% BSA in Tris buffer at 4 °C under constant shaking. After 6, 24, 72, 120, and 168 h, 20 µl solution was collected and frozen. BMP2 concentrations were then measured using the BMP2 ELISA development kit (Peprotech), according to manufacturer’s guidelines.

### Bioactivity assessment of released BMP2

The bioactivity of the released rhBMP2 was assessed by evaluating the ability of BMP2 to induce alkaline phosphatase activity (ALP), an early osteogenic differentiation marker^36,45^. C2C12 cells (American Type Culture Collection, Manassas, VA) were seeded in a 24-well plate, and cultured with DMEM supplemented with 10% fetal bovine serum (FBS, Gibco), 1% 200 mM l-glutamine and penicillin (100 U/ml, Gibco), streptomycin (100 µg/ml, Gibco) also referred as 1% P/S. After 24 h, cells were exposed to TG-PEG hydrogels placed into the upper chamber of the transwell. The hydrogels (20 μl) of different stiffness containing 2 μg of BMP2 were covered by 120 μl of DMEM media. After 7 days of incubation, ALP activity was measured using p-nitrophenylphosphate (Sigma) as a substrate as previously described^62^.

### Human bone marrow-derived stromal cells (hBMSCs)

hBMSCs were obtained from Prof. Ivan Martin at the University of Basel. They were isolated from bone marrow aspirates of healthy donors^63^ with informed consent from the local ethical committee (Ethics Committee of the University of Basel; Prof. Dr. Kummer; approval date 26/03/2007 Ref Number 78/07). hBMSCs were cultured at 37 °C in a humidified atmosphere at 5% CO_2_ using MEMα (with nucleosides, Gibco) supplemented with 10% FBS, 1% P/S, and fibroblast growth factor 2 (FGF-2, 5 ng/ml, PeproTech). Cells were passaged before reaching 90% confluency, and medium was changed every 2–3 days.

### hBMSCs proliferation

hBMSCs were encapsulated at 1.5 × 10^6^ cells per ml of TG-PEG hydrogel (15 µl) and cultured in MEMα with 10% FBS and 1% P/S. To assess proliferation, hydrogels were washed with PBS and adenosine triphosphate (ATP) was measured using the CellTiter-Glo® luminescent cell viability assay (Promega) following the manufacturer’s protocol. Total ATP at day 4, 8 and 12 was normalized to total ATP at d0 (right after encapsulation).

### Live and dead staining

hBMSCs were encapsulated at 1.5 × 10^6^ cells per ml of TG-PEG hydrogel (15 µl) and cultured in MEMα with 10% FBS and 1% P/S. After encapsulation, at day 4, day 8 and day 12, hydrogels were washed with PBS and incubated with 4 µM calcein and 2 µM ethidium bromide in PBS for 30 min at 37 °C. Gels were then washed with PBS, and directly imaged. Cells were imaged with Leica TCS SP5 confocal microscope.

### Immunocytochemistry of cultured hBMSCs

For fibronectin staining, mouse anti-fibronectin antibody (1/50, sc59826) was added to the cells while in culture for 7 h after 3 days post-encapsulation in TG-PEG hydrogels. Then, gels were washed 3× with PBS, and fixed in 4% paraformaldehyde for 30 min at room temperature (RT), followed by several washes of PBS. Next, gels were incubated with secondary antibody goat anti-mouse AF488 (1/200, ab150113), phalloidin-rhodamine (1:400) and 4′,6-diamidino-2-phenylindole (DAPI, 1 µg/ml) in 1% BSA in PBS at 4 °C overnight. Gels were then extensively washed in PBS, and finally imaged with Leica TCS SP5 confocal microscope.

### Calvarial defect model

This study adhered to the ARRIVE principles. All in vivo experiments were approved by the veterinary offices of the canton of Zurich (ethical license (Application No. ZH107/2012 and Application No. ZH86/2018)). They were conducted in accordance with the Swiss law of animal protection. 100 healthy 84–92 day old female Sprague–Dawley (SD) rats were obtained from Charles River Laboratories and used in this study. Under general anesthesia, 8 mm diameter craniotomies were created in the parietal bones of the skull using a trephine bur (Bien-Air, Bienne, Switzerland). Defects that showed signs of dura injuries were not used for transplantations. Per condition, at least n = 5 pre-formed sterile TG-PEG hydrogel discs (42.5 μl) with a diameter of 8 mm that contained 0 or 5 µg BMP2 were then placed in the defects. The TG-PEG hydrogel discs were produced in advance as previously described (in the section: 3D TG-PEG hydrogel formation). After placing the hydrogel into the defect, the skin was closed with sutures. 4 weeks after surgery animals were euthanized with CO_2_ (4 l/min). To ensure that animals were dead, additionally a bilateral pneumothorax was performed. Hydrogels that at explantation were found to be misplaced from the defect area and were found instead on top of murine bone, were discarded from the analysis to not confound the results.

### Micro-computed tomography analysis

Micro-computed tomography (microCT), image processing and analysis 3D imaging data were performed as described previously^64^. Briefly, after formalin fixation and equilibration in PBS, all bone defects were scanned in a microCT 40 (Scanco Medical AG) in high resolution-mode using an energy of 55 kVp and an intensity of 72 µA at 4 W. The resulting 10 µm voxel size images were reconstructed and a global threshold of 10% of the maximum grey value was applied to obtain segmented bone tissue structures in all samples. A 6 mm diameter cylindrical mask was manually placed in the center of the defect bone to ensure no pre-existing bone fragments were included in the quantifications. In these defined bone regions, the following bone parameters were evaluated in the newly formed bone area: bone volume (BV) providing the total volume, bone surface (BS) giving the total surface, bone connectivity density (Conn.D) representing the density of bone connections, and bone coverage calculated from high-resolution radiograph-like images derived from superior–inferior projections of the cranium. 3D reconstructions of the bone defect areas were obtained with the 3D Viewer plugin in ImageJ.

### Histological analysis

Bone samples were treated by sequential water substitution with ethanol, infiltration with xylene followed by methyl-methacrylate (MMA). Polymerization was carried out at 37 °C. The section were prepared (6.5 μm) from the middle of the defects and stained with Goldner trichrome as indicated previously^65^.

### Statistical analysis

In vitro data are reported as mean ± standard error, and in vivo data are reported as individual data points and means. All statistical analyses were performed using GraphPad Prism (version 8.0.0, GraphPad Software). Mean values were compared by one-way or two-way analysis of variance (ANOVA) followed by Tukey’s post hoc test for multiple comparisons. Statistical significance was accepted for P < 0.05, and reported as follows * P < 0.05, ** P < 0.01, *** P < 0.001, **** P < 0.0001. Further information is found in each figure legends. Additionally, for study groups of Figs. 5 and 6 with clearest differences, Post-hoc power analysis showed significance (using 0.05 alpha) between groups given the sample sized we analyzed. More specifically, in Fig. 5B the BMP2 effect in bone volume (BV) had 91.1% power calculation and in coverage attained 99.6% power with 0.05 alpha; in Fig. 6F to show the stiffness effect in connectivity density (Conn.D) a 99.9% power with 0.05 alpha was accomplished. Similarly, in Fig. 6H, stiffness effect in BV reached 94.6% power with 0.05 alpha, and stiffness effect in bone surface (BS) had 97.8%, 100%, and 99.5% power with 0.05 alpha, respectively in Fig. 6I. In Fig. 6J, degradability effect for Conn.D accomplished a power of 82%, while the stiffness effect was 83.1% and 82.9% all with 0.05 alpha. Ultimately, in Fig. 6K, coverage across stiffness reached a 78.3%, 91.4%, and 89.3% power with 0.05 alpha.

### Supplementary Information

Supplementary Figures.

## Data Availability

The datasets used and/or analysed during the current study are available from the corresponding author on reasonable request.
