# Supplementary material for: Enhanced bone regeneration in rat calvarial defects through BMP2 release from engineered poly(ethylene glycol) hydrogels
Source: Sci Rep. 2024 Feb 28;14:4916. doi: 10.1038/s41598-024-55411-z (PMC10901800; doi:10.1038/s41598-024-55411-z)
Supplement: Supplementary file 1 — Supplementary Figures. [file 41598_2024_55411_MOESM1_ESM.docx]

**Supplementary Data**

**Enhanced bone regeneration in rat calvarial defects through BMP2 release from engineered poly(ethylene glycol) hydrogels**

Queralt Vallmajo-Martin^1,2^, Christopher Millan^3^, Ralph Müller^4^, Franz E. Weber^5^, Martin Ehrbar*^1^ and Chafik Ghayor*^4^


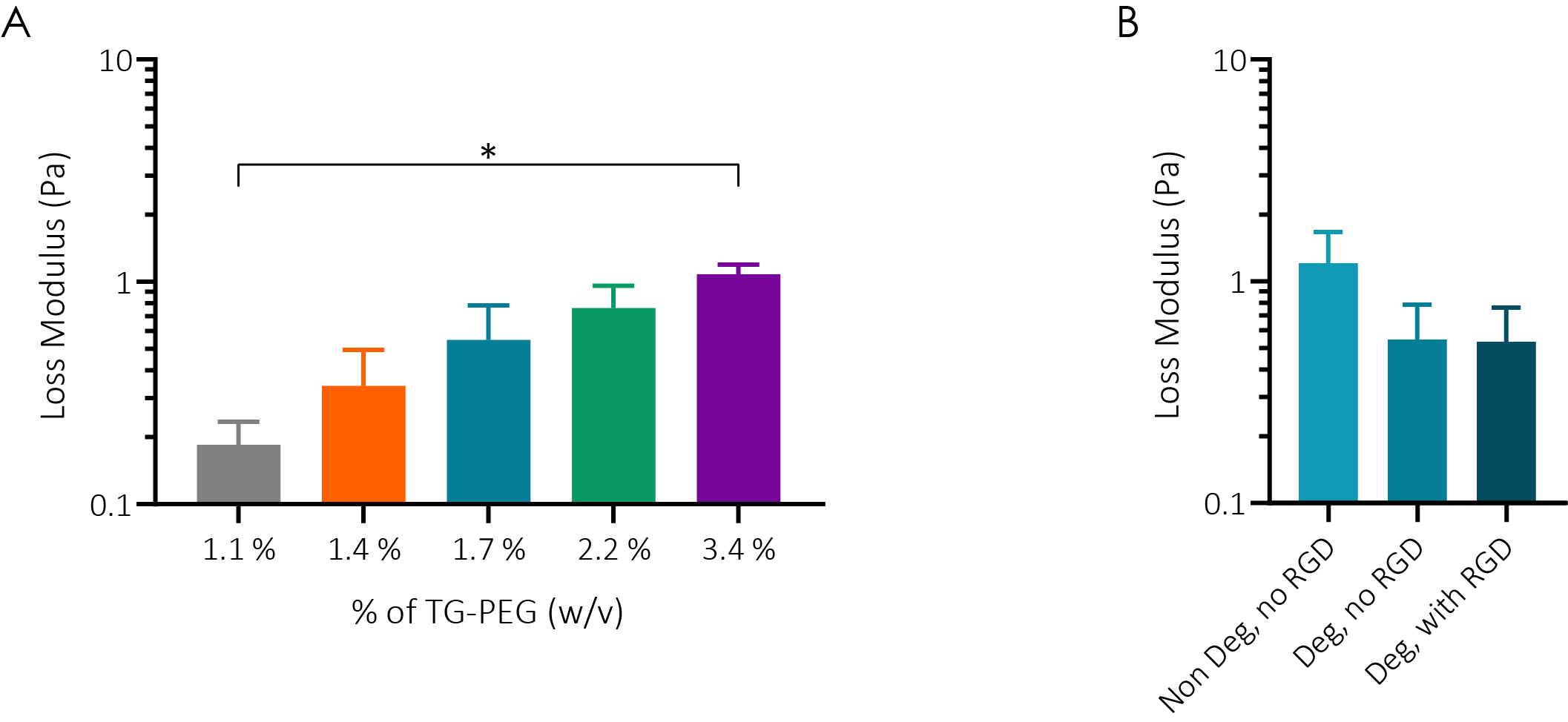


Figure S1**. Mechanical and physical characterization of TG-PEG hydrogels**

**a-b)** Loss moduli of hydrogels after reaching a plateau at 30 min **a)** at different polymer concentrations (n = 3) or **b)** at a fixed 1.7% (w/v) polymer concentration containing MMP_non-degradable_ (Non Deg) or MMP_sensitive_ (Deg) cross-links, and with or without RGD adhesion sites (n = 3). All data are reported as mean ± standard error. ANOVA with Tukey’s post hoc test * P < 0.05.


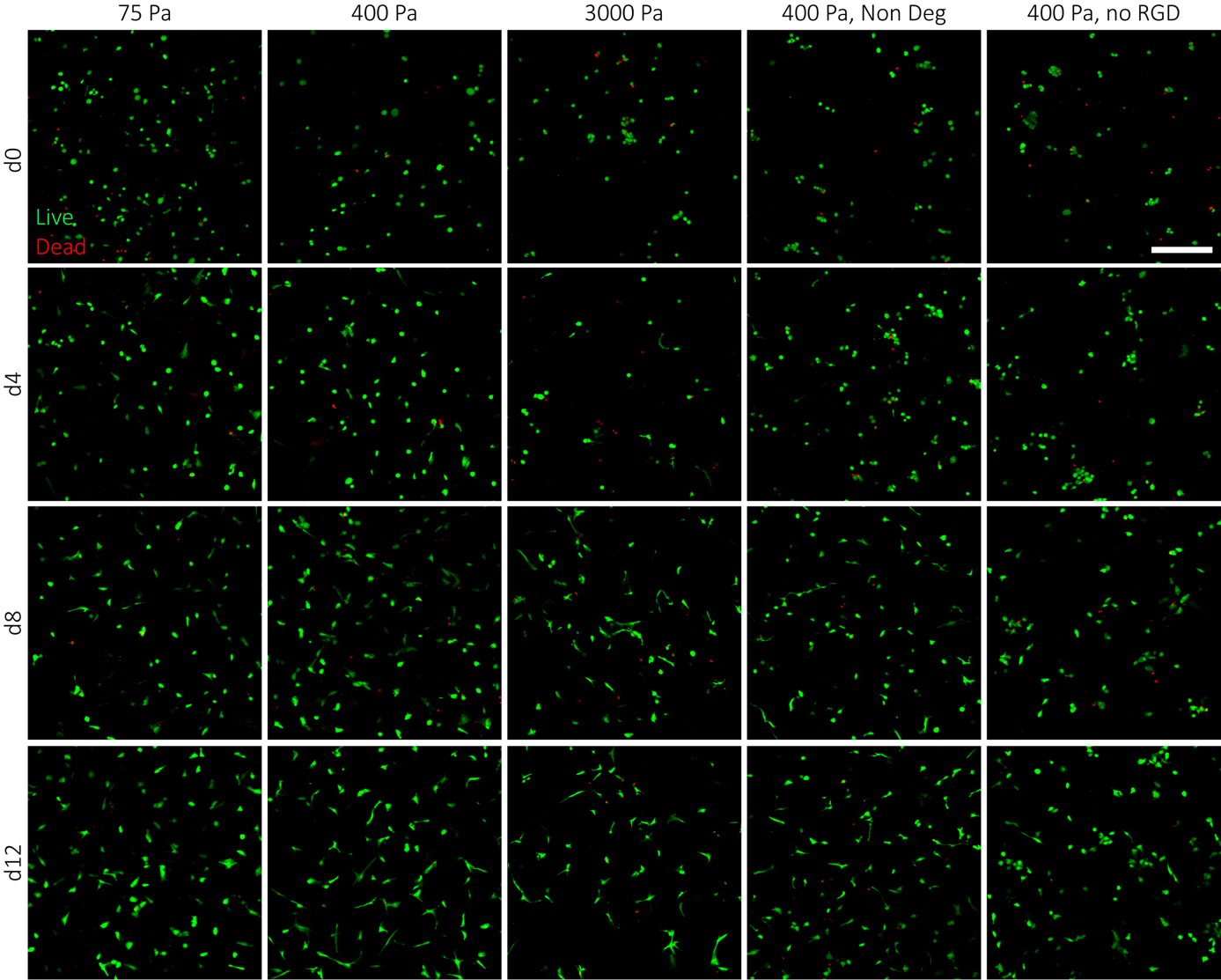


Figure S2**. hBMSCs viability in various TG-PEG hydrogel conditions**

Human bone marrow-derived stromal cells (hBMSCs) were encapsulated at 1.5·10^6^ cells per ml of TG-PEG hydrogels. TG-PEG hydrogels at different stiffnesses (75, 450 or 3000 Pa) containing MMP_sensitive_ degradable sites and RGD cell adhesion sites were tested, as well as TG-PEG gels at a fixed stiffness of 450 Pa lacking either the MMP_sensitive_ site (Non Deg) or the RGD peptide (no RGD). Representative images of live and dead staining on hBMSCs over time encapsulated in different hydrogel conditions (live cells stained by calcein are in green, while dead cells stained in ethidium bromide are in red; scale bar: 200 µm).
